# Supplementary material for: A game theoretic treatment of contagion in trade networks
Source: PLoS Comput Biol. 2025 Dec 26;21(12):e1013845. doi: 10.1371/journal.pcbi.1013845 (PMC12788693; doi:10.1371/journal.pcbi.1013845)
Supplement: S1 Appendix — (PDF) [file pcbi.1013845.s001.pdf]

1. A Necessary Lemma

Lemma 1 (An elementary analysis lemma)**.** *Suppose x_k is a real sequence in $\mathbb{R}^n$ which converges to x. Let $a, b : \mathbb{R}^n \to \mathbb{R}$ which are both continuous at x, with $|b(x)| < 1$. Then the sequence given by the recursive form $y_k = a(x_k) + b(x_k)y_{k-1}$ has a limit*

$$\lim y_k = \frac{a(x)}{1 - b(x)} \tag{1}$$

so long as a and b are such that y_k is non-negative.

Proof. First I argue that y_k must be a bounded sequence if $a(x_k)$ and $b(x_k)$ are both bounded sequences with $b(x) < 1$. We know that $b(x_k) < 1 - \delta$ whenever $k > K_b$ for some $\delta > 0$ and some K_b. We also know that $a(x_k) < A$ for some A. Now compare the sequence $z_k = A + (1-\delta)z_{k-1}$ to the sequence y_k. If $y_k - z_k < 0$, the same inequality will hold for the next terms

$$y_{k+1} - z_{k+1} < (1-\delta)(y_k - z_k) + (b_1 - (1-\delta))y_k < 0$$

Let z_0 be such that $z_{K_b} > y_{K_b}$ (This is possible because the recurrence relation is bijective). Therefore $y_k < z_k$ for all $k > K_b$ and, because z_k is obviously bounded, y_k must also be bounded.

Now consider the shifted sequence so that the limit is 0. That is, consider $e_k = |y_k - \frac{a(x)}{1-b(x)}|$. Our sequence e_k is also a bounded sequence $|e_k| < M$.

$$
\begin{aligned}
y_k - \frac{a(x)}{1-b(x)} &= a(x_k) + b(x_k)y_k - \frac{a(x)}{1-b(x)} \\
&= a(x_k) + b(x_k)\left(y_{k-1} - \frac{a(x)}{1-b(x)}\right) - \frac{b(x_k)a(x)}{1-b(x)} + \frac{a(x)}{1-b(x)} \\
&= b(x_k)e_{k-1} + a(x_k) - a(x)\frac{1-b(x_k)}{1-b(x)} \\
e_k &\le |b(x_k)|e_{k-1} + \left|a(x_k) - a(x)\frac{1-b(x_k)}{1-b(x)}\right|
\end{aligned}
$$

Let $\varepsilon > 0$ and we will show that there is a K so that $k > K \implies e_k < \varepsilon$. Observe that e_k is nonnegative for all k and that given any δ, there is a K_1 so that $|b(x_k)| < 1 - \delta$ for all $k > K_1$ because $b(x_k) \to b(x) < 1$. Moreover, because it is a combination of continuous functions, as $z \to x$,

$$
a(z) - a(x)\frac{1-b(z)}{1-b(x)} \to a(x) - a(x)\frac{1-b(x)}{1-b(x)} = 0.
$$

Thus, for any $\eta > 0$ there is a K_2 so that $\left|a(x_k) - a(x)\frac{1-b(x_k)}{1-b(x)}\right| < \eta$.

Now let $K = \max\{K_1, K_2\}$. If we consider the sequence

$$
d_k = (1-\delta)d_{k-1} + \eta
$$

we know that if $e_K \le d_K$ then $e_k \le d_k$ for all $k > K$ (by the standard comparison argument in the first paragraph of this proof). Clearly, because $(1-\delta) < 1$, this sequence converges to the limit $d = \frac{\eta}{1-(1-\delta)} = \frac{\eta}{\delta}$. To complete the proof. Select any reasonable δ and get the associated K_1. Then let $\eta < \frac{\delta\epsilon}{2}$

and get the associated K_2. For these selections of δ and η, and resulting $K = \max\{K_1, K_2\}$ we know that for any d_0, $d_k \to \frac{\varepsilon}{2}$ and thus there is a K_{d_0} so that $d_k < \varepsilon$ for all $k > K_d$. This K_{d_0} depends on our choice for d_0. Select a d_0 so that $d_K > M$ (This is possible because the recurrence relation is bijective) and the result is that $e_k < d_k$ for all $k > K$ so $e_k < \epsilon$ for all $k > K_{d_0}$. Thus we have shown that $e_k \to 0$ which completes the proof. $\square$
