# Supplementary material for: A game theoretic treatment of contagion in trade networks
Source: PLoS Comput Biol. 2025 Dec 26;21(12):e1013845. doi: 10.1371/journal.pcbi.1013845 (PMC12788693; doi:10.1371/journal.pcbi.1013845)
Supplement: Attachment — Submitted filename: Review_PLOS_Computational_Biology (2).pdf [file pcbi.1013845.s002.pdf]

REVIEW OF: A GAME THEORETIC TREATMENT OF CONTAGION IN TRADE NETWORKS

Overview

The paper is clearly written. The main message is that based on some convergence analysis, the stochastic model of a trade network with an infectious agent leads to a unique stationary point from which payoffs can be analytically calculated. This allows calculation of best responses and hence the study of Nash equilibria. Some qualitative analysis is done on a toy model.

I have pointed out a few details in the mathematical analysis which deserve attention. Please check over the notation carefully for any additional inconsistencies such as the one mentioned below in Theorem 1.

Technical

(-) Between lines 231-232, I believe the last term on the right should be

$$\frac{\sum_{i=1}^{m} s_{i,m+1}(1-(r_{m+1,m+1}-\sum_{i=1}^{m} s_{i,m+1})^k)}{1-r_{m+1,m+1}+\sum_{i=1}^{m} s_{i,m+1}}$$

rather than

$$\frac{(1-\sum_{i=1}^{m} s_{i,m+1})^k}{1-r_{m+1,m+1}+\sum_{i=1}^{m} s_{i,m+1}}$$

in order to satisfy the recurrence relation above.

(-) Before the presentation of Theorem 1, it is announced that the index is changed for clarity so that there is a total of n nodes. But then the theorem still references the map T with domain $1^m \times [0,1]^n$, so no re-indexing has taken place.

(-) In lines $430-31$, the "Naïve risk" is defined to be $1-\prod_{i=1}^{n}(1-p_i^*)$, "which is the probability that at least one individual is infected." But this calculation must assume independence of infection at each node, at stationarity, in order to multiply the probabilities $(1-p_i^*)$. This independence was never shown, which is an issue that should be addressed.

(-) Appendix A contains the following lemma:

Lemma 1 (An elementary analysis lemma) Suppose $\vec{x}_k$ is a real sequence which converges to $\vec{x}$. Let $a, b : \mathbb{R}^n \to \mathbb{R}$ which are both continuous at $\vec{x}$, with $|b(\vec{x})| < 1$. Then the sequence given by the recursive form $y_k = a(\vec{x}_k) + b(\vec{x}_k)y_{k-1}$ has a limit

$$\lim y_k = \frac{a(\vec{x})}{1-b(\vec{x})}$$

so long as a and b are such that $y_k > 0$ for all k.

The proof of this lemma looks very careful/rigorous, but it runs to six pages, which takes a lot of energy to process. Could you write the proof more simply using the Banach fixed-point theorem, something like this?

Observe that

$$\underbrace{\left|y_k - \frac{a(\vec{x})}{1-b(\vec{x})}\right|}_{\Delta_k} = \left|a(\vec{x}_k) + b(\vec{x}_k)y_{k-1} - \frac{a(\vec{x})}{1-b(\vec{x})}\right|$$

$$= \left|b(\vec{x}_k)\left(y_{k-1} - \frac{a(\vec{x})}{1-b(\vec{x})}\right) + a(\vec{x}_k) - a(\vec{x})\left(\frac{1-b(\vec{x}_k)}{1-b(\vec{x})}\right)\right|$$

$$\leq |b(\vec{x}_k)|\underbrace{\left|y_{k-1} - \frac{a(\vec{x})}{1-b(\vec{x})}\right|}_{\Delta_{k-1}} + \underbrace{\left|a(\vec{x}_k) - a(\vec{x})\left(\frac{1-b(\vec{x}_k)}{1-b(\vec{x})}\right)\right|}_{\varepsilon_k}$$

So $\Delta_k \leq |b(\vec{x}_k)|\Delta_{k-1} + \varepsilon_k$. Now argue that $\Delta_k \to 0$. Here is one suggestion:

We are assuming $b(\vec{x}_k) \to b(\vec{x})$ with $|b(\vec{x})| < 1$, and $a(\vec{x}_k) \to a(\vec{x})$. This means that $\varepsilon_k \to 0$. Now there must exist some $0 < \beta < 1$, $\alpha > 0$, and K so that for all $k \geq K$, we have $|b(\vec{x}_k)| < \beta$ and $\varepsilon_k < \alpha$.

So the values Δ_k are dominated by the values Δ'_k defined for all $k \geq K$ by

$$\Delta'_K = \Delta_K, \quad \Delta'_k = \beta\Delta'_{k-1} + \alpha$$

The map $z \mapsto \beta z + \alpha$ is a contracting map. By the Banach fixed-point theorem, $\Delta'_k \to \alpha/(1-\beta)$. In particular, the limsup of the values Δ_k is at most $\alpha/(1-\beta)$. Since $\alpha > 0$ was arbitrary, $\Delta_k \to 0$. In other words,

$$y_k \to \frac{a(\vec{x})}{1-b(\vec{x})}$$

Typos etc

I make no promise of completeness here:

(-) The sentence in lines 165-166 is confusing due to a grammatical error.

(-) Line 182: "directly *from*"

(-) Broken sentence in line 299.

(-) Line 310: "There *exists* R"

(-) Line 320: "$\subset\subset$"

(-) Lines 664-667 contain typos.
