# Supplementary material for: A game theoretic treatment of contagion in trade networks
Source: PLoS Comput Biol. 2025 Dec 26;21(12):e1013845. doi: 10.1371/journal.pcbi.1013845 (PMC12788693; doi:10.1371/journal.pcbi.1013845)
Supplement: Attachment — Submitted filename: Review_PCB-Mcalister.pdf [file pcbi.1013845.s003.pdf]

The work incorporates several factors such as economic trading in wildlife and strategic choices in investing in safety measures to ascertain how these factors affect the spread of contagion in acyclic, hierarchical wildlife trade networks. Contagion is spread only during the trading process and not just through contacts between the traders. The work is focused on obtaining Nash equilibrium solutions which would be the best response given the strategies of other members of the population.

The first part is primary involved in obtaining Nash equilibrium through calculation of the equilibrium infection probabilities p*. This allows them to determine the payoff to player i, given the strategies of all the remaining players and hence compute the best response to that strategy set X_{-i}. Formal mathematical proofs are given for these parts.

The toy model in section 5 considers contagion spread and equilibrium strategies in 3-layered acyclic network with different structures corresponding to different symmetry configurations and different degrees of monopoly implemented at the level of the distributor (layer 2). The aim is to show how the equilibrium strategy, long-term equilibrium infection probability and contagion risks vary with different network structures.

The work is well-motivated with clearly stated goals, and the work addresses an important problem in epidemiology associated with wildlife trade. Key differences with the previous literature have been clearly highlighted. However, the style of writing (particularly sections 2-5) makes the paper more appropriate for a journal of mathematical biology than Plos Computational Biology. The application of computational methods (as specified in the Scope of PCB) in the analysis is rather limited in this article and restricted to the toy model discussed in section 5. The style of presentation would be difficult for non-mathematicians to follow. I have not come across such a presentation style in PCB articles. Even though I find the overall message interesting, I unfortunately cannot recommend it for publication in Plos Computational Biology for the above reasons.

Specific feedback, that I hope will benefit the authors, are given below.

- What are the special features of this model that make it specifically relevant to study infection risks in animal trade networks and not just any other type of contagion spread in a generic trade network? It would be helpful if the authors clarify that aspect in the introduction.
- The article does not provide any interpretation of the different terms in the expression for the payoff to strategy *x_i*. It would be helpful to the reader if the interpretation of each term is given. For instance, for payoff contributions from downstream interactions, the largest benefit to individual '*i*' corresponds to the partner strategy *x_j*=0 (i.e. a partner who is a cheater and makes no investment in safety measures) but it is modulated with the probability (*w_ij(1-x_jI_i)*) of interaction with the j'th partner and vanishes when *i* is infected (*i_i=1*) and *x_j=1* i.e. the j'th partner (buyer) who has maximally invested in safety measures will not interact with an infected seller. This makes trading between infected sellers and cautious buyers less likely lowering the benefit of infected sellers. Such interpretations will improve readability.
- I did not quite understand the rationale behind payoff contribution from the intrinsic benefit term *x_i(x_i-1)(1-I_i)*. This suggests that if the i'th individual is not infected, the intrinsic payoff depends on the strategy but is maximum (zero) for x_1=0,1 and minimum

(-0.25) for $x\_i=0.5$. This suggests that intrinsic payoff is maximum only if there is no investment ($x\_i=0$) or full investment ($x\_i=1$) in safety. Moderate investment in protection ($x\_i \sim 0.5$) is disfavoured. What is the rationale behind this ?

- The intrinsic benefit weight is not defined in the main text. Yet Fig.5 plots the changes in equilibrium strategy as the intrinsic benefit weight is varied.
- There are several instances where the definitions are given in mathematical terms without interpretations or explanation. Perhaps this is a common style of presentation in mathematics journals, but it makes the paper difficult to read and understand for non-mathematicians. As an example, eq. 7 is written without any explanation or interpretation of the different terms that make up the payoff to strategy x_i when the remaining strategies are X_{-i}.

Minor comments

Pg22, line 366 should read "....each player has a one dimensional....."

Pg23, line371: To say that "player i's strategy increases...." sounds strange. I understand that the strategy x_i is defined in terms of the degree of investment in safety measures which increases.
