# Supplementary material for: A game theoretic treatment of contagion in trade networks
Source: PLoS Comput Biol. 2025 Dec 26;21(12):e1013845. doi: 10.1371/journal.pcbi.1013845 (PMC12788693; doi:10.1371/journal.pcbi.1013845)
Supplement: Attachment — Submitted filename: WildlifeTradeNetworksResponseToReviewers.pdf [file pcbi.1013845.s004.pdf]

A Game Theoretic Treatment of Contagion in Trade Networks: Response to Reviewers

John McAlister

October 2025

Thank you for the insightful revisions you both offered on this manuscript. I have included a point by point response to the reviews below. You each mentioned helpful ways that this manuscript could be changed to be more accessible to the more biological audience of PCB. Throughout the manuscript, we have added in more description of and more connections to the application area in order to correct this. A more specific point by point response to your revisions is included below.

Reviewer 1

- Between lines 231-232, I believe the last term on the right should be

$$\frac{\sum_{i=1}^{m} s_{i,m+1}(1-(r_{m+1,m+1}-\sum_{i=1}^{m} s_{i,m+1})^k)}{1-(r_{m+1,m+1}-\sum_{i=1}^{m} s_{i,m+1})}$$

  rather than

$$\frac{1-(\sum_{i=1}^{m} s_{i,m+1})^k}{1-r_{m+1,m+1}+\sum_{i=1}^{m} s_{i,m+1}}$$

  in order to satisfy the recurrence relation above.

  Thank you for this note. The version that we included in our manuscript was incorrect. Your solution is correct. However to make the following step more clear, we have modified the argument to use the linearity of the difference equation to our advantage. For clarity we use the temporary variable $a = \sum_{i=1}^{m} s_{i,m+1}$ and $b = r_{m+1,m+1} - \sum_{i=1}^{m} s_{i,m+1}$ and $x_k = [T^{(k)}p]_{m-1}$. The recurrence relation becomes $x_{k+1} = a + bx_k$ or $x_{k+1} - bx_k = a$. The solution to the homogeneous equation is $x_k = Cb^k$ for some C and a particular solution to the inhomogeneous problem is $x_k = \frac{a}{1-b}$ for all k. The general solution therefore is $x_k = Cb^k + \frac{a}{1-b}$. We solve for C when $k = 0$ to get $C = [p_{m+1}] - \frac{a}{1-b}$.

  Although we write the general solution as

$$[T^{(k)}p]_{m+1} = C\left(r_{m+1,m+1} - \sum_{i=1}^{m} s_{i,m+1}\right)^k + \frac{\sum_{i=1}^{m} s_{i,m+1}}{1-r_{m+1,m+1}+\sum_{i=1}^{m} s_{i,m+1}}.$$

The equivalence to your solution can be seen simply by plugging in the expression for C.

- Before the presentation of Theorem 1, it is announced that the index is changed for clarity so that there is a total of n nodes. But then the theorem still references the map T with domain $1^m \times [0,1]^n$, so no re-indexing has taken place.

  Thank you for catching this, Because reindexing does not add more clarity, we no longer reindex and let the indices be from the range $1, 2, ..., m, m + 1, ..., m + n$.

- In lines 430-31, the "Naïve risk" is defined to be $1 - \prod_{i=1}^{n}(1 - p_i^*)$, "which is the probability that at least one individual is infected." But this calculation must assume independence of infection at each node, at stationarity, in order to multiply the probabilities $(1 - p_i^*)$. This independence was never shown, which is an issue that should be addressed.

  Thank you very much for pointing out this oversight. You are correct that what we claimed to be the probability that anyone in the trade network is infected is incorrect exactly because the infections in the network are not independent. Because this measure of risk is quickly abandoned in favor of the more sophisticated weighted risk, we have revised the manuscript to note that the naive risk is merely the upper bound for the probability that at least one player in the trade network is infected.

- *regarding Lemma 1* The proof of this lemma looks very careful/rigorous, but it runs to six pages, which takes a lot of energy to process. Could you write the proof more simply using the Banach fixed-point theorem?

  Thank you for giving this alternate proof. The argument you gave is correct and indeed more concise than our argument. We have changed the argument of Lemma 1 to follow your proof with the necessary inclusion that y_k is bounded under the assumptions of the lemma. We have included more detail in each step and, instead of appealing to the BFPT, we treat the existence and uniqueness of the limit of $z_k = a + bz_{k-1}$ as a standard fact with $|b| < 1$.

- The sentence in lines 165-166 is confusing due to a grammatical error.

  Thank you for catching this error. We changed the sentence to read: The probability of player i interacting with player j when j is infected ($a_{i,j}(X,1)$) is given as an entry in the matrix W by $w_{i+m,j+m}$.

- Line 320 "$\subset\subset$"

  Thank you for bringing this to our attention. We originally used this notation to mean a compact subset but we agree that this notation is not common enough among the audience of PLOS Comp. Bio. to use it without explanation. We changed this line to explicitly state $\Omega \subset \mathbb{R}^{s \cdot m}$ and that Ω is compact.

- Other Typos:

  Thank you for catching these typos. We have corrected them and examined the manuscript for other remaining errors.

Reviewer 2

- What are the special features of this model that make it specifically relevant to study infection risks in animal trade networks and not just any other type of contagion spread in a generic trade network? It would be helpful if the authors clarify that aspect in the introduction.

  Thank you for this insightful comment. The goal of this paper is to put forward a model that is potent to study the spread of any type of contagion in a trade network. The focus on the wildlife trade network comes from the fact that it is an easy to imagine example and from the fact that for a single stakeholder in a wildlife trade network, the entire stock can become infected rather quickly which gives the model more predictive power. Paragraphs explaining this have been added in the introduction and in the model explanation.

- The article does not provide any interpretation of the different terms in the expression for the payoff to strategy x_i. It would be helpful to the reader if the interpretation of each term is given.

  Thank you for this note. As a toy model, we did not want to risk giving to much meaning to each term lest we make the reader think they are earnest attempts to be predictive. However, we agree that an explanation, with the level of detail that you offered in your review, would go a long way in improving understanding of the model. We have added such explanations in section 5.

- I did not quite understand the rationale behind payoff contribution from the intrinsic benefit term $x_i(x_i - 1)(1 - I_i)$. This suggests that if the i'th individual is not infected, the intrinsic payoff depends on the strategy but is maximum (zero) for $x_1 = 0, 1$ and minimum (-0.25) for $x_i = 0.5$. This suggests that intrinsic payoff is maximum only if there is no investment ($x_i = 0$) or full investment ($x_i = 1$) in safety. Moderate investment in protection (x_i 0.5) is disfavoured. What is the rationale behind this ?

  Thank you so much for catching this mistake, This should read $x_i(1 - x)i)(1 - I_i)$. You are absolutely right that flipping the second monomial the other way reverses the expected behavior. If you examine the code which was made available in the data availability statement you will see that it was written correctly in the simulation. We corrected it in the text so now it is correct every where. We are truly thankful that you caught this crucial typo.

- The intrinsic benefit weight is not defined in the main text. Yet Fig.5 plots the changes in equilibrium strategy as the intrinsic benefit weight is varied.

  Thank you again for pointing out this oversight. The parameter being changed is now explicitly introduced in the description of the toy model and the explination is made more clear in the discussion of the that numerical experiment in subsection 5.3

- There are several instances where the definitions are given in mathematical terms without interpretations or explanation. Perhaps this is a common style of presentation in mathematics journals, but it makes the paper difficult to read and understand for non mathematicians

  Thank you for this note. We appreciate this insight and have gone through the entire text to make sure that whenever definitions are given in mathematical terms, they are also explained in the context of the application area. We hope that this, along with the other edits we have made to strengthen the connection to the application area make the manuscript easier better suited for the audience of PCB.

- minor comments

  Thank you for these comments, we have corrected these errors in the texts and spent time ensuring there are no other such typos.
