# Supplementary material for: A game theoretic treatment of contagion in trade networks
Source: PLoS Comput Biol. 2025 Dec 26;21(12):e1013845. doi: 10.1371/journal.pcbi.1013845 (PMC12788693; doi:10.1371/journal.pcbi.1013845)
Supplement: Attachment — Submitted filename: GameTheoreticWTNResponseToReviewers.pdf [file pcbi.1013845.s005.pdf]

A Game Theoretic Treatment of Contagion in Trade Networks: Response to Reviewers

John McAlister

December 2025

Reviewer 1

- The naive risk is used as an upper bound for the probability of infection at some node. Of course this is intuitive since we expect infections to be positively correlated rather than negatively correlated, but it's not immediately obvious mathematically why it would be an upper bound. If you don't want to justify this mathematically, I would state it only as a non-rigorous heuristic, or else remove it entirely.

  Thank you for mentioning this. We have added a section in the text justifying this assertion through elementary conditional probability.

Reviewer 2

- L130-131: If 'i' is the focal player (consumer) and j is the seller, then shouldn't line 130 read as, "...the benefit of the player 'j' is given b_{ji}..." The statement can perhaps be framed in a different way to enhance clarity..... "The cost of such an interaction for the focal player 'i' is given by $c_i, j(x_i, x_j)$ when 'i' buys a good from an upstream 'j' player; and the benefit for the seller j is given by $b_j, i(x_i, x_j)$."

  We thank the reviewers for catching this indexing error. It is correct that the indices should be switched. We have adapted the language here to make it more clear.

- L248-250 has been written in red and then crossed out. Why?

  In the version of the revision with changes tracked, this line was crossed out to indicate that it was removed. The line does not appear in the version of the revision without changed tracked

- L446-450: I did not understand the meaning of the statement in line 449 that says "..the intrinsic benefit is not measured in a qualitative measure"enjoyment"." I thought that the intrinsic benefit is being quantified through the strategy (x_i) of the focal player and her infection status (I_i).

Thank you for pointing out that this is unclear. We have changed the text and added some to now say:

"One of the major issues with game theoretic models with intrinsic benefit is that determining how important the intrinsic benefit is relative to the extrinsic factors can be very difficult, especially when the intrinsic benefit is measured in a qualitative measure like "enjoyment" while the extrinsic factors are attached to a currency. From a modeling perspective, this is an issue of unit analysis. If $d_i(x_i, I)$ from equation (1) has units of "enjoyment" while $c_{i,j}(x_i, x_j)$ and $b_{i,j}(x_i, x_j)$ have units of currency, we much include a conversion factor which relates the currency to the enjoyment."

The benefit to player i depends on x_i and I_i but it is given by some function of these two $d_i(x_i, I_i)$ is the toy model, f is quite simple but we still must consider how the units of d_i compare to the units of $c_{i,j}$ and $b_{i,j}$.

- L479 should read: "...and the other two buy from both s1 and s2 evenly."

  Thank you for catching this typo
